# Novel Requirement for Staphylococcal Cell Wall-Anchored Protein SasD in Pulmonary Infection

**DOI:** 10.1128/spectrum.01645-22

**Published:** 2022-08-30

**Authors:** Jennifer A. Grousd, Brooke P. Dresden, Abigail M. Riesmeyer, Vaughn S. Cooper, Jennifer M. Bomberger, Anthony R. Richardson, John F. Alcorn

**Affiliations:** a Department of Immunology, University of Pittsburgh grid.21925.3dgrid.471408.egrid.21925.3d , Pittsburgh, Pennsylvania, USA; b Department of Pediatrics, UPMC Children’s Hospital of Pittsburgh, Pittsburgh, Pennsylvania, USA; c Department of Microbiology & Molecular Genetics, University of Pittsburgh grid.21925.3dgrid.471408.egrid.21925.3d , Pittsburgh, Pennsylvania, USA; University of Florida

**Keywords:** influenza, lung, MRSA, pneumonia, macrophages

## Abstract

Staphylococcus aureus can complicate preceding viral infections, including influenza virus. A bacterial infection combined with a preceding viral infection, known as superinfection, leads to worse outcomes than a single infection. Most of the pulmonary infection literature focuses on the changes in immune responses to bacteria between homeostatic and virally infected lungs. However, it is unclear how much of an influence bacterial virulence factors have in single or superinfection. Staphylococcal species express a broad range of cell wall-anchored proteins (CWAs) that have roles in host adhesion, nutrient acquisition, and immune evasion. We screened the importance of these CWAs using mutants lacking individual CWAs *in vivo* in both bacterial pneumonia and influenza superinfection. In bacterial pneumonia, the lack of individual CWAs leads to various decreases in bacterial burden, lung damage, and immune infiltration into the lung. However, the presence of a preceding influenza infection partially abrogates the requirement for CWAs. In the screen, we found that the uncharacterized CWA S. aureus surface protein D (SasD) induced changes in both inflammatory and homeostatic lung markers. We further characterized a SasD mutant (sasD A50.1) in the context of pneumonia. Mice infected with sasD A50.1 have decreased bacterial burden, inflammatory responses, and mortality compared to wild-type S. aureus. Mice also have reduced levels of interleukin-1β (IL-1β), likely derived from macrophages. Reductions in IL-1β transcript levels as well as increased macrophage viability point at differences in cell death pathways. These data identify a novel virulence factor for S. aureus that influences inflammatory signaling within the lung.

**IMPORTANCE** Staphylococcus aureus is a common commensal bacterium that can cause severe infections, such as pneumonia. In the lung, viral infections increase the risk of staphylococcal pneumonia, leading to combined infections known as superinfections. The most common virus associated with S. aureus pneumonia is influenza, and superinfections lead to worse patient outcomes than either infection alone. While there is much known about how the immune system differs between healthy and virally infected lungs, the role of bacterial virulence factors in single and superinfection is less understood. The significance of our research is identifying bacterial components that play a role in the initiation of lung injury, which could lead to future therapies to prevent pulmonary single or superinfection with S. aureus.

## INTRODUCTION

Respiratory viral infections can be complicated by bacterial pneumonia, leading to increased rates of morbidity and mortality compared to the viral infection alone. Staphylococcus aureus has been shown to complicate several viral infections, such as influenza, respiratory syncytial virus, and rhinovirus (1). This is also seen in the current viral pandemic with COVID-19, with one study finding a mortality rate of over 60% for those infected with both SARS-CoV-2 and S. aureus (2). While S. aureus is considered to be a common commensal, it can cause severe disease such as endocarditis, bacteremia, sepsis, and death (3). Combined with a preexisting viral infection (colloquially referred to as superinfection), it is unsurprising that outcomes in patients that are superinfected with S. aureus are worse than either bacterial or viral infection alone. The virus most commonly associated with S. aureus is influenza. Influenza is a seasonal respiratory virus that causes an estimated 294,000 to 518,000 deaths worldwide each year (4). Since 2009, S. aureus has been the primary contributor to influenza bacterial superinfections (5–8).

Preceding viral infections increase the susceptibility to S. aureus infection through three main mechanisms: (i) modifying the expression of host proteins involved in S. aureus adhesion or internalization, (ii) synergism of viral- and bacterial-induced epithelial invasion and damage, and (iii) reduced clearance of the bacteria by altering the immune response (1, 9–13). A large body of literature exists that is focused on the immunological differences in antibacterial immunity that occur with a preceding viral infection (12–14). In general, the antiviral response inhibits the clearance of a bacterial infection through various mechanisms (9–13). There are also physiological differences that occur due to viral infection that may increase susceptibility to secondary infections. Many of the bacteria known to cause superinfection are nasal commensals, and inflammation from influenza has been shown to increase dissemination from the nasopharynx to the lung (10, 15). The virus itself, and the immune response to the virus, can lead to epithelial and endothelial damage, which could lead to increased nutrient resources as well as potentially expose cryptic receptors for bacterial adherence to cells or basement membrane components (9, 10, 16). Influenza neuraminidase and wound healing responses can alter the cellular expression of receptors on cells, which may act as adhesion sites for bacteria (9, 10, 17, 18). Once the bacteria are attached, bacterial toxins could synergize with the virus to cause further damage and inflammation in the lung, potentially leading to the increased morbidity and death in superinfection (9, 10, 19).

Few studies have focused on the bacterial side of single or superinfections. For S. aureus specifically, the roles of few virulence factors have been described within the lung, even within the context of S. aureus pneumonia. Because the superinfection literature suggests that viral infection can influence bacterial adherence, we explored surface proteins of S. aureus, collectively known as the cell wall-anchored proteins (CWAs), since many of these proteins have known roles in adhesion (20, 21). S. aureus can express up to 24 CWAs, with the most prevalent subfamily known as microbial surface component recognizing adhesive matrix molecule (MSCRAMM) proteins (20, 21). CWAs are covalently attached to the cell wall via the sortase A enzyme, which recognizes the cell wall sorting motif LPXTG (20, 22–24). Because these are surface-exposed proteins, they are in contact with the host and have a variety of known functions, such as host adhesion, biofilm formation, immune evasion, and nutrient acquisition for both colonization and invasive infection (20, 21). Some CWAs have been described to play a part in nasal colonization (20, 21, 25). Since these CWA proteins play an important part in S. aureus colonization and infection, we decided to screen several CWA members in the lung in both bacterial pneumonia and influenza superinfection.

## RESULTS

### Screening cell wall-anchored protein mutants during bacterial pneumonia and influenza superinfection.

**FIG 1:**
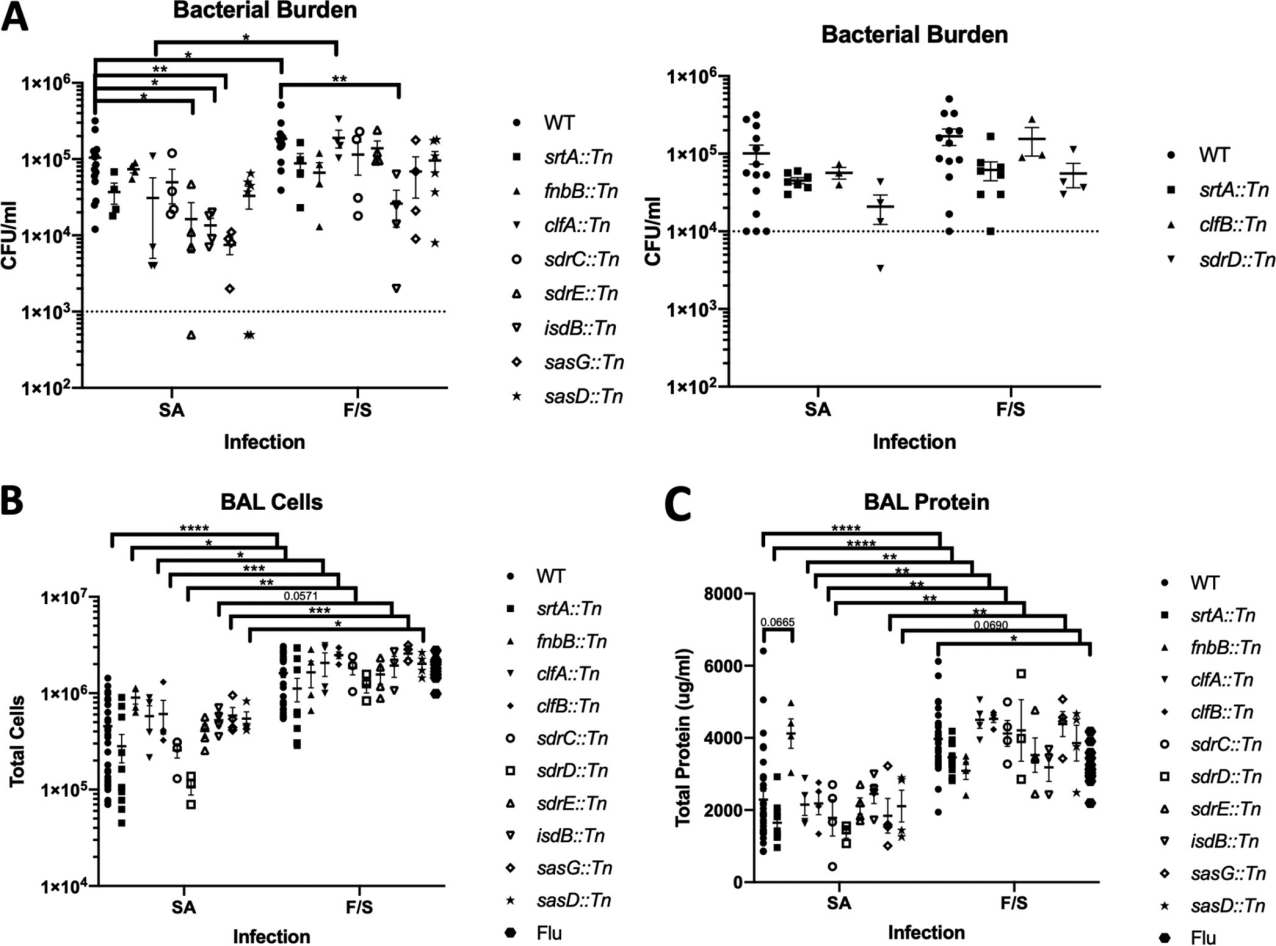
Differential impact of S. aureus CWA mutants in bacterial pneumonia and influenza superinfection. Mice were inoculated with PBS or 100 PFU of influenza on day 0 and 6 days later were infected with PBS or 1 × 10^8^ CFU of WT MRSA or a strain lacking individual CWA (see graphs) and were harvested 24 h later. (A) Bacterial burden in bacterial pneumonia (SA) or influenza superinfection (FS) 24 h post-MRSA infection. Mice with undetectable CFU were graphed as half of the limit of detection. (B and C) Total cells (B) and total protein (C) in the bronchoalveolar lavage (BAL) fluid in bacterial pneumonia (SA) or influenza superinfection (FS). Statistics were tested by two-way ANOVA with Sidak’s multiple-comparison correction. *, *P* < 0.05; **, *P* < 0.01; ***, *P* < 0.001; ****, *P* < 0.0001. *N* = 2 to 4; combination of several experiments; data are graphed as the mean ± standard error of the mean (SEM). srtA, sortase A; fnbB, fibronectin binding protein B; clfA/B, clumping factor A/B; sdrC/D/E, serine-aspartate repeat containing protein C/D/E; isdB, iron-regulated surface determinant B; sasD/G, S. aureus surface protein D/G.

Since cell wall-anchored proteins (CWAs) are exposed on the cell surface of S. aureus, we hypothesized that these proteins may be playing a role in colonization and/or infection in the lung. We screened nine CWA mutants (*fnbB*::*Tn*, *clfA*::*Tn*, *clfB*::*Tn*, *sdrC*::*Tn*, *sdrD*::*Tn*, *sdrE*::*Tn*, *isdB*::*Tn*, *sasG*::*Tn*, and *sasD*::*Tn*) and the corresponding wild-type (WT) strain JE2 in the context of bacterial pneumonia and influenza superinfection. We also included a sortase A (*srtA*::*Tn*) mutant, the enzyme responsible for attaching these CWAs to the cell wall of S. aureus. In terms of bacterial burden within the lung (Fig. 1A), lacking individual CWA proteins during bacterial pneumonia led to various decreases in burden compared to the WT strain. The differences in bacterial burden did not appear to be due to differences in *in vitro* growth rates of the various mutants (see Fig. S1A and B in the supplemental material). Interestingly, the sortase A mutant did not have a significant decrease in bacterial burden. The mutant lacking SasG (S. aureus surface protein G; *sasG*::*Tn*) had the largest decrease in burden during bacterial pneumonia. During superinfection, mutants had increased burden versus single infection, although only the WT and ClfA mutant (clumping factor A; *clfA*::*Tn*) were significantly increased. The mutant lacking IsdB (iron-regulated surface determinant protein B; *isdB*::*Tn*) was the only mutant that had significantly decreased burden in both bacterial pneumonia and superinfection. Lacking S. aureus CWAs did not impact viral burden in the lung (Fig. S1C).

To look at lung inflammation with CWA mutants, we examined the number of cells in the airway via bronchoalveolar lavage (BAL) (Fig. 1B). Cellular immune infiltrates in the lung varied based on the mutant. During superinfection, almost all mutants had significantly higher numbers of BAL cells in the airways. The number of BAL cells during superinfection were similar to influenza-alone levels, suggesting that primary influenza infection is the main driver of immune infiltrate during superinfection. To look at acute lung injury and leak, we measured total protein in the BAL specimen (Fig. 1C). Although not as variable, the level of protein in the BAL specimen during bacterial pneumonia varied based on the mutant. Interestingly, the mutant lacking FnbB (fibronectin binding protein B; *fnbB*::*Tn*) had the highest level of protein in the BAL specimen in bacterial pneumonia. Lung leak also significantly increased during superinfection compared to bacterial pneumonia. Only the WT had significantly increased BAL protein during superinfection compared to influenza alone.

### Immune responses to CWA mutants in bacterial pneumonia and influenza superinfection.

**FIG 2:**
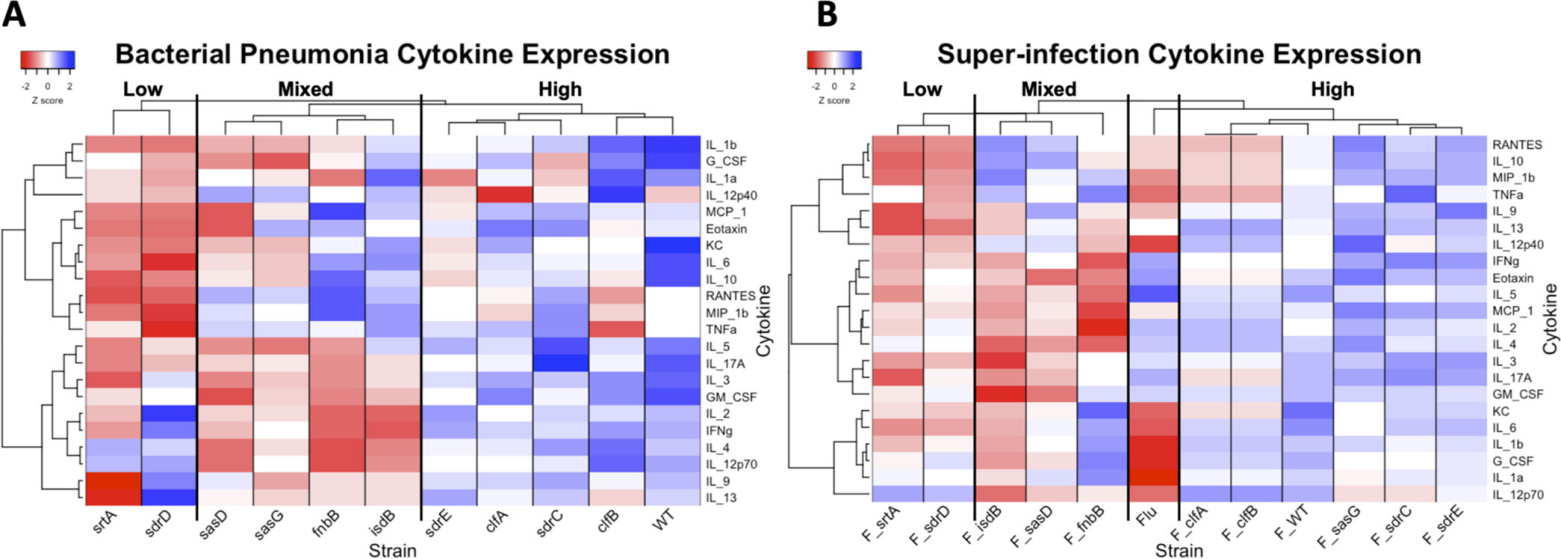
CWA mutant induced cytokine expression in bacterial pneumonia and influenza superinfection. (A) Cytokine expression in bacterial pneumonia. There are three clusters (from left to right): low inflammation, mixed inflammation, and high inflammation. (B) Cytokine expression in superinfection. There are four clusters (from left to right): low inflammation, mixed inflammation, influenza alone (Flu), and high inflammation. Cytokines were measured via multiplex analyses of lung homogenate. For each cytokine, average values for each MRSA strain were log transformed and converted to Z scores. Heatmap clustering was performed by cytokine using the Pearson correlation and graphed using the heatmap.2 function of the gplots package in R. srtA, sortase A; fnbB, fibronectin binding protein B; clfA/B, clumping factor A/B; sdrC/D/E, serine-aspartate repeat containing protein C/D/E; isdB, iron-regulated surface determinant B; sasD/G, S. aureus surface protein D/G.

Next, we examined the inflammatory response to CWA mutants via cytokines in lung homogenate. To determine if immune signatures were similar in CWA subfamilies, we visualized the cytokine data by clustering analyses (Fig. 2A and B). In bacterial pneumonia, there were three clusters of inflammatory responses, which we termed low inflammation, mixed inflammation, and high inflammation (Fig. 2A). Unsurprisingly, the sortase A mutant, which lacks all CWAs on the cell surface, had the lowest level of cytokine induction. *srtA*::*Tn* had significant decreases in type 2 cytokines interleukin-9 (IL-9) (*P* < 0.0001) and IL-13 (*P* = 0.0025), but not IL-4 and IL-5. This was not driven by IL-33 expression, as *srtA*::*Tn* trended toward increased IL-33 expression via quantitative PCR (qPCR) (*P* = 0.0710) (data not shown). The other mutant in the low-inflammation cluster was *sdrD*::*Tn* (serine aspartate repeat containing protein D), which had higher levels of expression of type 1 and type 2 cytokines than *srtA*::*Tn.* The mutants found in the mixed-inflammation cluster typically had higher levels of innate immunity cytokines and chemokines but lower levels of type 1, 2, and 17 cytokines. The high-inflammation cluster, which contained the WT strain as well as most Clf and Sdr members, had the highest levels of cytokines. During superinfection, the clustering of CWAs by cytokine expression was very similar to bacterial pneumonia (Fig. 2B). Again, the strains cluster into three groups distinct from influenza alone, and the only mutant that switched clusters was *sasG*::*Tn*.

### Characterization of SasD during bacterial pneumonia.

**FIG 3:**
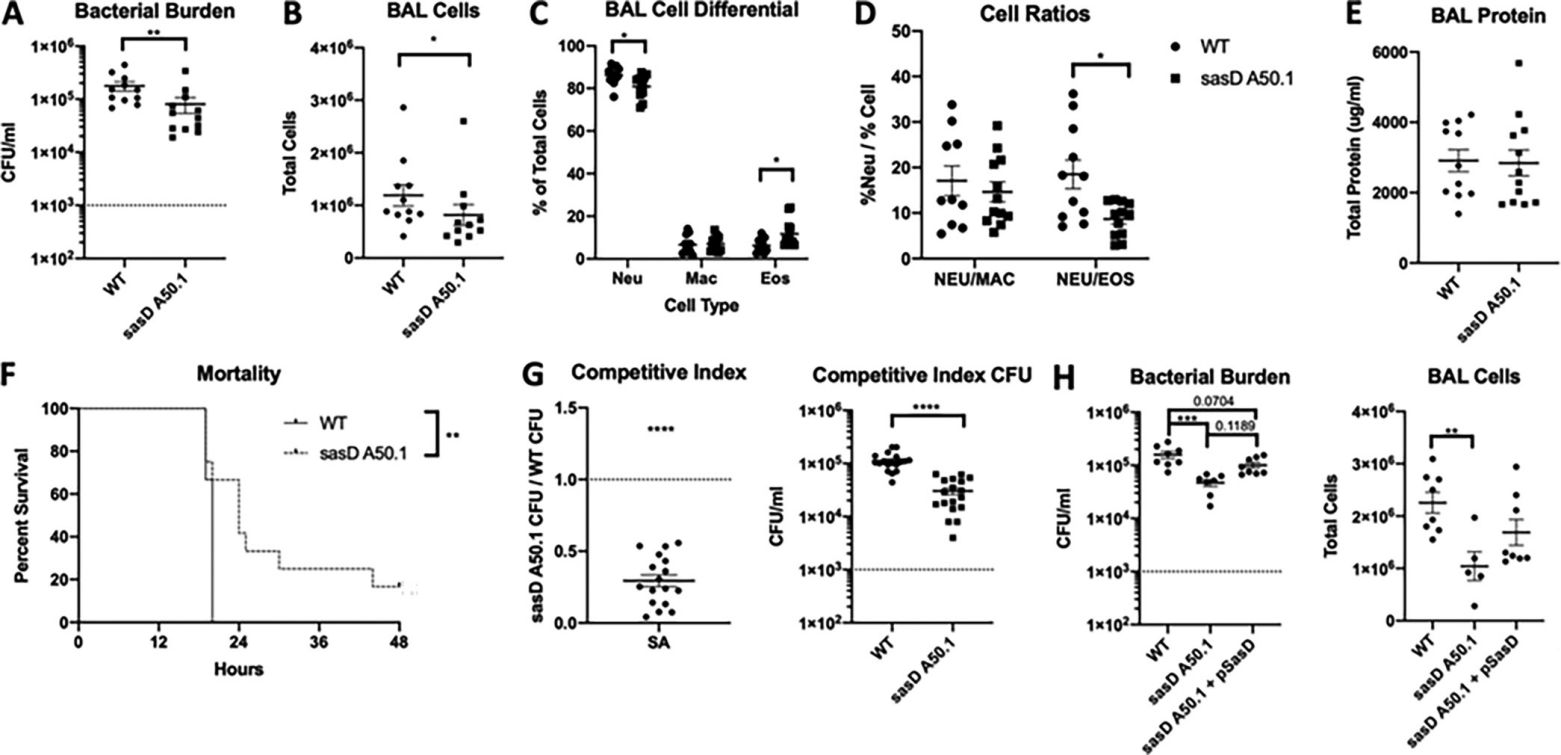
SasD impacts S. aureus bacterial pneumonia outcomes. (A to E) Mice were infected with 1 × 10^8^ CFU WT MRSA or MRSA lacking SasD (sasD A50.1) for 24 h. (A) Bacterial burden in mice infected with MRSA for 24 h. (B) Total cells in the bronchoalveolar lavage (BAL) fluid. (C and D) Cell differentials (C) and neutrophil cell ratios (D) of BAL cells. (E) Total protein in the BAL fluid. (F) Mice were infected with a lethal dose (2 × 10^8^ CFU) of WT or MRSA lacking SasD (sasD A50.1). (G) Competitive index of WT and mutant sasD A50.1 MRSA in the lung. Mice were infected with a 1:1 ratio of WT:sasD A50.1 for a total dose of 1 × 10^8^ CFU for 24 h. Whole lungs were collected in 2 mL PBS and homogenized and plated for CFU with and without antibiotic selection. The competitive index is calculated as the ratio of mutant CFU:WT CFU at 24 h postinfection (hpi). (H) Bacterial burden and total cells in the BAL fluid in mice infected with MRSA for 24 h. Statistics were tested by student Mann-Whitney (A, B, D, and G), two-way ANOVA with Sidak’s multiple comparisons (C), log-ranked Mantel Cox test (F), one sample *t* test with H_0_ set to 1 (1:1 ratio of mutant:WT) (G), and one-way ANOVA with Sidak’s multiple comparisons (H). *, *P* < 0.05; **, *P* < 0.01; ***, *P* < 0.001; ****, *P* < 0.0001. *N* = 4 to 8; combination of several experiments; data graphed as mean ± SEM.

The data suggest that preceding influenza abrogates the requirement for S. aureus CWAs in terms of bacterial burden, BAL cellularity, and protein leak. For this reason, we next focused on single S. aureus infection to interrogate CWAs. We identified that the mutant lacking SasD (S. aureus surface protein D; *sasD*::*Tn*) induced reduced levels of cytokines G-CSF, CXCL1, MCP-1, and IL-1β compared to the WT strain during bacterial pneumonia (Fig. S2A). Additionally, we also saw increased levels of epithelial and lung homeostasis marker gene expression, including tight junction protein 1 (*tjp1*) and mucin 5b (*muc5b*) (Fig. S2B). This suggested to us that this mutant may be causing less inflammation in the lung, leading to improved epithelial and lung function. Very little is known about SasD, and to our knowledge, there has been no *in vivo* characterization of this CWA. Therefore, we decided to characterize this mutant in the context of bacterial pneumonia. To eliminate the potential for unknown mutations in the transposon mutant, we transduced the mutant into the JE2 strain, creating the strain sasD A50.1. This strain had no difference in *in vitro* growth compared to the WT strain (Fig. S3A and B). At 24 h postinfection, mice infected with sasD A50.1 had reduced bacterial burden and immune infiltrate in the BAL fluid (Fig. 3A and B). Infection with sasD A50.1 led to a decrease and increase in the percentage of neutrophils and eosinophils, respectively. While there were no changes in genes related to neutrophil function (Fig. S3C), we did see a 50% reduction in the neutrophil:eosinophil ratio in the lung (Fig. 3C and D). The change in bacterial burden and immune cell infiltrate did not lead to differences in acute lung injury by BAL protein (Fig. 3E) or histological score (Fig. S3D and E). However, we did see significant changes in transcripts related to lung homeostasis (Fig. S3F). Additionally, we saw a significant delay in mortality in mice infected with sasD A50.1 compared to WT S. aureus (Fig. 3F). The changes seen 24 h postinfection in mice infected with sasD A50.1 may be because of a decrease in survival of the mutant in the lung, as this mutant had a reduced competitive index when mice were infected with a 1:1 ratio of mutant:WT bacteria (Fig. 3G). Complementation of SasD in the sasD A50.1 mutant strain increased both bacterial burden and BAL cells to similar levels as the WT strain (Fig. 3H).

**FIG 4:**
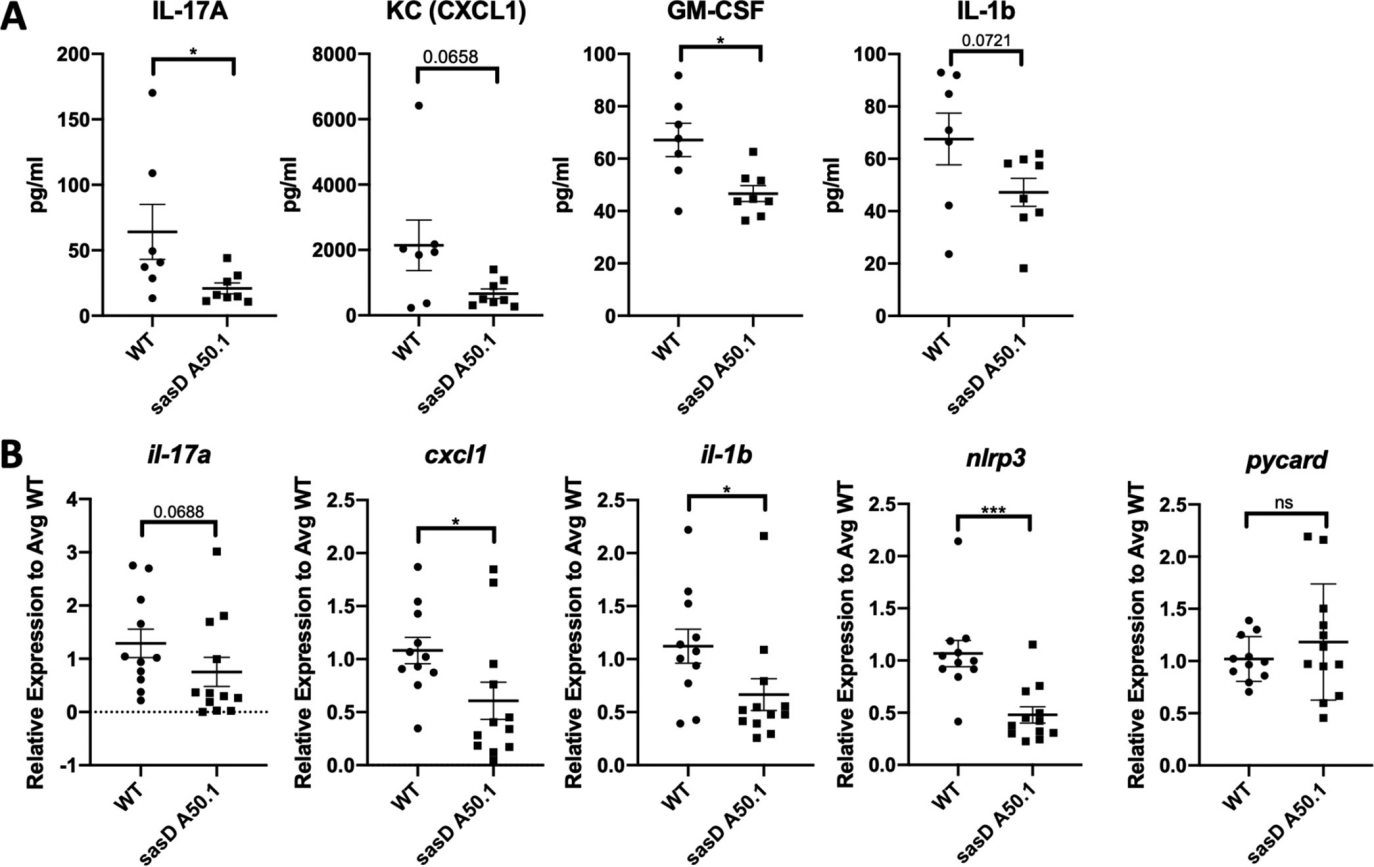
SasD increases inflammation in mice infected with MRSA. Mice were infected with 1 × 10^8^ CFU WT MRSA or MRSA lacking SasD (sasD A50.1) for 24 h. (A) Cytokine protein levels in lung homogenate. (B) Gene expression levels of cytokines and inflammasome components relative to average WT levels in the lung. Statistics were done by Mann-Whitney test. *, *P* < 0.05; ***, *P* < 0.001. *N* = 4; combination of several experiments; data graphed as mean ± SEM.

To evaluate the inflammatory state within the lung, we looked at cytokine expression in the lung 24 h postinfection. Similar to the original screen, we saw decreases in protein levels of IL-17A, CXCL1, granulocyte-macrophage colony-stimulating factor (GM-CSF), and IL-1β in mice infected with sasD A50.1 (Fig. 4A). We also saw a decrease in transcript levels for IL-17A, CXCL1, and IL-1β (Fig. 4B). Because we saw a decrease in IL-1β and S. aureus is known to induce the NLRP3 (NOD-, LRR- and pyrin domain-containing protein 3) inflammasome (26), we also looked at inflammasome components. We saw a significant decrease in NLRP3 transcript, but not the general inflammasome adaptor ASC (apoptosis-associated speck-like protein containing C-terminal caspase recruitment domain [CARD]; *pycard*) (Fig. 4B). S. aureus alpha-toxin is a known inducer of the NLRP3 inflammasome (26). It is possible that sasD A50.1 may have impaired alpha-toxin production. However, we did not see differences in alpha-toxin activity assayed via hemolysis of blood agar in either strain (data not shown).

### Changes in inflammation occur early during infection with sasD A50.1.

**FIG 5:**
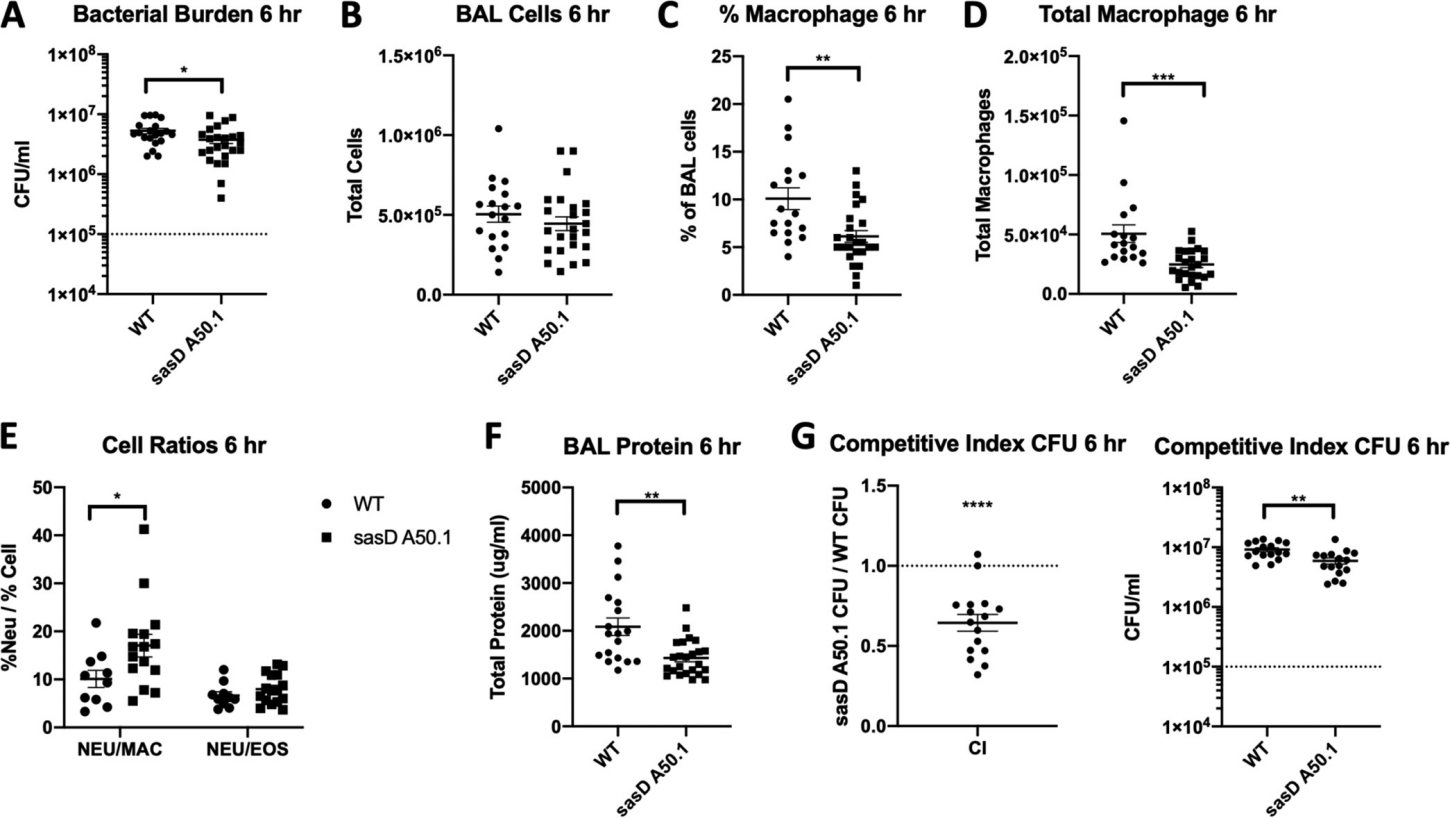
SasD impacts initiation of host defense against MRSA in bacterial pneumonia. (A to F) Mice were infected with 1 × 10^8^ CFU WT MRSA or MRSA lacking SasD (sasD A50.1) for 6 h. (A) Bacterial burden in mice infected with MRSA for 6 h. (B) Total cells in the bronchoalveolar lavage fluid. (C and D) Percentage (C) and total number (D) of macrophages in the BAL fluid. (E) Neutrophil cell ratios in the BAL fluid. (F) Total protein in the BAL fluid. (G) Competitive index of WT and mutant sasD MRSA in the lung. Male and female mice were infected with a 1:1 ratio of WT:sasD A50.1 for a total dose of 1 × 10^8^ CFU for 6 h. Whole lungs were collected in 2 mL PBS and homogenized and plated for CFU with and without antibiotic selection. The competitive index is calculated as the ratio of mutant CFU:WT CFU at 6 hpi. Statistics were tested by student Mann-Whitney (A, C, D, F, and G), two-way ANOVA with Sidak’s multiple comparisons (E), and one sample *t* test with H_0_ set to 1 (1:1 ratio of mutant:WT) (G). *, *P* < 0.05; **, *P* < 0.01; ***, *P* < 0.001; ****, *P* < 0.0001. *N* = 8; combination of several experiments; data graphed as mean ± SEM.

Mice infected with sasD A50.1 for 6 h also had a reduction in bacterial burden compared with the WT (Fig. 5A). While the total number of immune cells recruited into the airway was not different between WT- and sasD A50.1-infected animals (Fig. 5B), we did see significant decreases in the percentages and total numbers of macrophages in the BAL specimens (Fig. 5C and D). This increase in the neutrophil:macrophage ratio in the lung during this early time point (Fig. 5E) may be associated with less neutrophil recruitment later during infection. Unlike 24 h postinfection, we did see a significant difference in acute lung injury via protein in the BAL specimens (Fig. 5F), and there was a significant difference in peribronchial inflammation via histology (Fig. S4A and B). However, we did not see changes in lung homeostasis transcripts (Fig. S4C). We also saw a survival defect of this mutant early on in infection via competitive index (Fig. 5G), which does not appear to be due to differences in early recruited neutrophil function (Fig. S4D).

**FIG 6:**
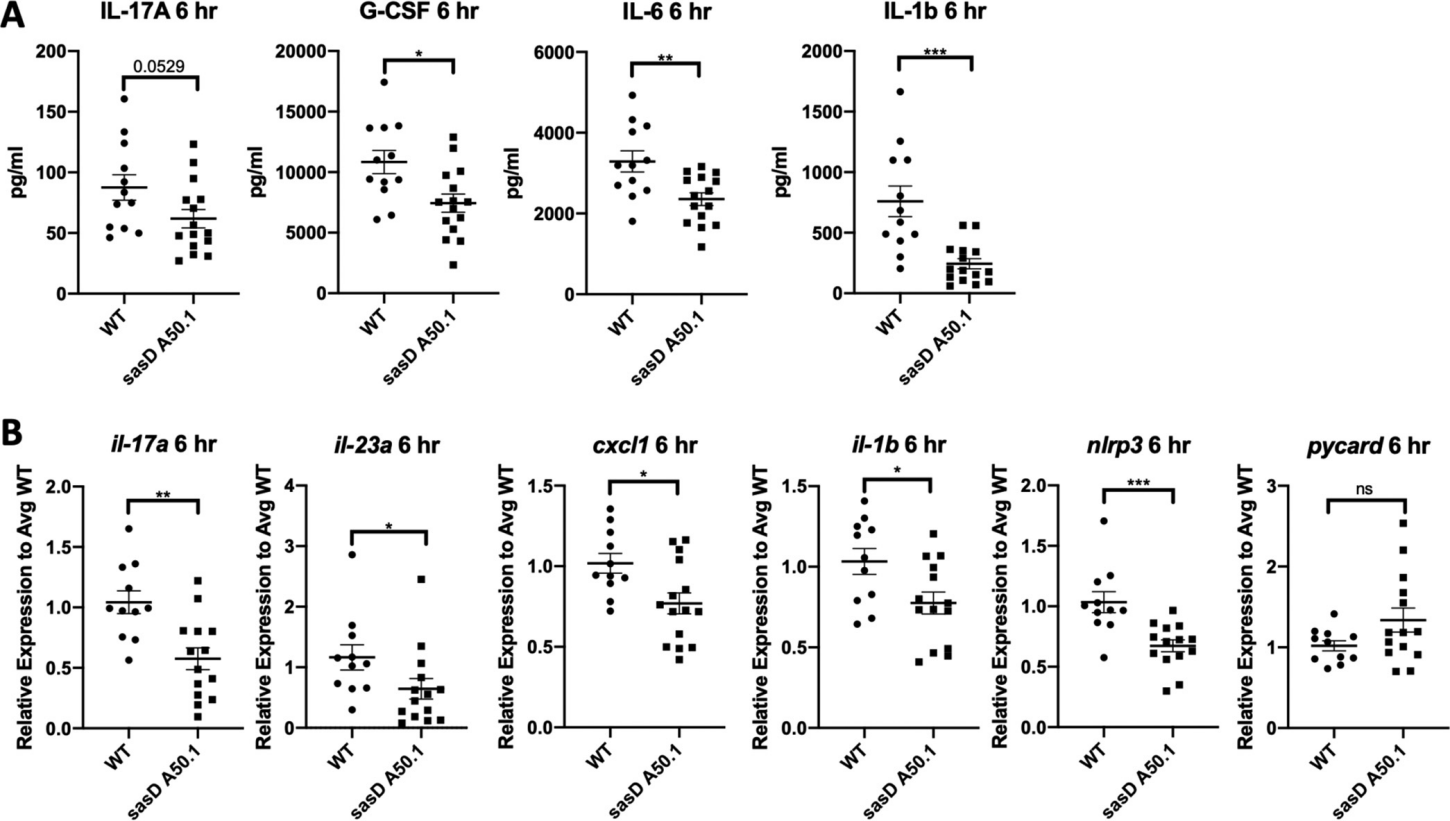
SasD is required for early inflammation during infection with MRSA. Mice were infected with 1 × 10^8^ CFU WT MRSA or MRSA lacking SasD (sasD A50.1) for 6 h. (A) Cytokine protein levels in lung homogenate. (B) Gene expression levels of cytokines and inflammasome components relative to average WT levels. Statistics were done by student Mann-Whitney test. *, *P* < 0.05; **, *P* < 0.01; ***, *P* < 0.001. *N* = 8; combination of several experiments; data graphed as mean ± SEM.

The inflammatory response to sasD A50.1 at 6 h postinfection was very similar to that at 24 h postinfection (Fig. 6). Again, we saw reductions in IL-17A and IL-1β with the addition of a decrease in G-CSF and IL-6 (Fig. 6A). By transcript levels, we saw reductions in both IL-17A and IL-23A (Fig. 6B), suggesting that the antibacterial immunity response to sasD A50.1 is not as robust compared to that of the WT at this time point, potentially due to the difference in survival between the two strains (Fig. 5G). We also saw a reduction in inflammasome component NLRP3 but not ASC (Fig. 6B).

### Early macrophage interactions with sasD A50.1.

**FIG 7:**
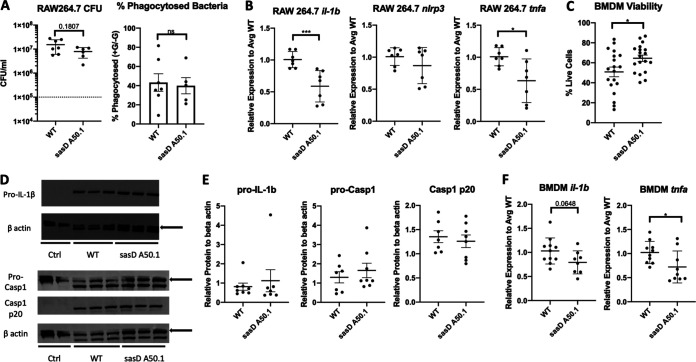
SasD increases macrophage inflammation and decreases survival. (A and B) RAW264.7 macrophages were infected with WT or sasD A50.1 MRSA for 3 h at an MOI of 10. Macrophages were infected for 1 h in the absence of antibiotics; the medium was then replaced with antibiotic- and serum-free medium with or without gentamicin for 1 h and changed to antibiotic free medium. CFU and transcript graphs are shown without gentamicin conditions. (A) Bacterial burden and percent phagocytosed bacteria in RAW264.7 macrophages. The percent phagocytosed bacteria is calculated by the following equation: ([average CFU with gentamicin]/[average CFU without gentamicin]) · 100. (B) Gene expression in RAW264.7 macrophages infected with WT or sasD A50.1 MRSA for 3 h. (C to F) Bone marrow-derived macrophages (BMDMs) were infected with WT or sasD A50.1 MRSA for 3 h at an MOI of 50 in the absence of antibiotics. C. Viability measured by trypan blue staining of BMDMs 3 h postinfection. (D and E) Representative images (D) and quantification of Western blot analyses (E) of BMDM levels of IL-1β and caspase 1. One to three wells were combined per sample, and protein levels are normalized to beta actin in each sample. Arrows denote which band was used for quantification. (F) Gene expression in BMDM macrophages infected with WT or sasD A50.1 MRSA for 3 h. Statistics were tested by Mann-Whitney test. *, *P* < 0.05; ***, *P* < 0.001. *N* = 4 to 7; combination of several experiments; data graphed as mean ± SEM.

Because of the differences seen in macrophages early on during infection, we looked at early macrophage interactions with sasD A50.1. Using the macrophage cell line RAW264.7, we saw a significant reduction in transcript expression of IL-1β and tumor necrosis factor alpha (TNF-α) despite no difference in bacterial burden or number of phagocytosed bacteria (Fig. 7A and B). In bone marrow-derived macrophages (BMDMs), infection with sasD A50.1 led to an increase in percent viability compared to WT-infected BMDMs at 3 h postinfection (Fig. 7C). At this time point we did not see any differences in pro-IL-1β or caspase 1 activity (Fig. 7D and E). However, we did see similar changes in IL-1β and TNF-α in BMDMs via qPCR compared with results in RAW264.7 cells (Fig. 7F).

## DISCUSSION

The majority of the viral superinfection literature focuses on the differences in immune responses between bacterial pneumonia and influenza bacterial superinfection. It is well documented that preceding influenza greatly impairs the antibacterial response within the lung (12, 13). Few studies have examined bacterial factors in single or superinfection in the lung. The literature suggests that increased inflammation and tissue damage lead to increased adhesion within the lung, contributing to increases in bacterial burden (9, 10). However, to our knowledge, there has been no specific testing of bacterial adhesion components *in vivo* during single or viral superinfection in the lung. Most studies that have investigated S. aureus virulence factors in the lung have focused on secreted toxins, such as the alpha-toxin (19, 27–30). While toxin-mediated damage contributes to lung pathology, the alpha-toxin has been shown to decrease adhesion to lung epithelial cells (31). Thus, we wanted to determine if proteins with known adhesion properties influenced the outcomes of single or superinfection.

Our data support the finding that changes due to influenza infection are the primary driver of superinfection, with influenza increasing bacterial burden, immune recruitment, and acute lung injury seen in the model. Interestingly, regardless of what CWA was removed, influenza appeared to “level the playing field” for the mutants, with endpoints being much higher and more tightly grouped in superinfection than in bacterial pneumonia alone. S. aureus strains seen in superinfected individuals are less virulent and more closely related to nasal colonizing strains than those strains found in bacterial pneumonia patients (32). This is likely due to the increased inflammation and damage within lung as well as a more dysregulated immune response during superinfection leading to less aggressive colonizing strains taking hold in the lung. However, viral-bacterial synergism is likely adding to this phenomenon, as influenza can increase both internalization and adhesion of bacteria within the lung (33, 34). This is not specific to influenza, as the same phenomenon is seen in rhinovirus-S. aureus superinfections (1, 35).

We saw more variability in the endpoints studied during bacterial pneumonia, likely because adhesion in the lung is more difficult in a homeostatic state. SasG has a known role in biofilm formation (36, 37), which may explain the decrease in burden seen in bacterial pneumonia. SasG has also been shown to adhere to human desquamated nasal epithelial cells via an unknown ligand (38), so it unclear if lacking SasG would have a pronounced impact on murine lung cell adhesion. IsdB is the receptor for hemoglobin and part of the heme acquisition system to attain iron, an important bacterial nutrient (39). While this protein has higher affinity for human hemoglobin, it still plays a role in murine models (39, 40). Thus, it is unsurprising that lacking this CWA had an impact on bacterial survival in both bacterial pneumonia and influenza superinfection. In bacterial pneumonia, *fnbB*::*Tn* had elevated protein in the BAL fluid, which may be caused by the high bacterial burden and immune cell infiltrate. FnbB, along with FnbA (not tested in this study), has been shown to play a role in invasion into nonprofessional phagocytes via fibronectin-integrin α_5_β_1_ interactions (41–43). This phenomenon has been shown *in vitro* for alveolar epithelium, and an FnbB deletion mutant was found to have increased protein leak in a rat model of pneumonia (44). This suggests that internalization of S. aureus, and subsequent immune evasion, may reduce inflammation in the lung. SdrD is known to play a role during nasal colonization as well as help promote survival of S. aureus in the blood (45–47). Lacking this CWA may allow for reduced a number of immune cells during bacterial pneumonia, as well as lower cytokine expression.

CWAs are known to bind to several proteins within the host, such as fibrinogen and fibronectin (21). In this study, we did not explore bacterial adhesion to specific ligands, but it is likely a combination of several ligands, as described at other host sites, such as the nose (48). CWAs also have overlapping ligands, such as ClfA, ClfB, FnbA, and FnbB, all binding fibrinogen (21). Because we only looked at single CWA mutants, some of the functions of these proteins in bacterial pneumonia and superinfection could be masked.

Even though the CWA mutants had more clear phenotypes in bacterial pneumonia compared to superinfection, the cytokine signature in both settings appears to be driven by the expression of these CWAs. The mutants found in each cluster were consistent in both bacterial pneumonia and superinfection, with the exception of *sasG*::*Tn*. This suggests that while a majority of the inflammation in the lung is driven by influenza, at least some part of the immune response is shaped by the presence of these CWAs on the cell surface of the bacteria. As SasG has a known role in biofilm formation and influenza is known to induce dissemination of S. aureus biofilms (15, 36, 37), this effect could influence how the immune system reacts to this mutant. A majority of the MSCRAMM proteins (*clfA*::*Tn*, *clfB*::*Tn*, *sdrC*::*Tn*, *sdrE*::*Tn*) cluster together in the high inflammation cluster. This is what we expected to find, as these proteins have similar domains used for ligand binding and this may influence the immune response (22). ClfA has been shown to be a T cell activator driving Th1 and Th17 activation (49). While we did not see any significant changes in IL-2 or gamma interferon (IFN-γ), we did see a nearly significant decrease in IL-17A (*P* = 0.0571) with the *clfA*::*Tn* mutant. Unsurprisingly, the sortase A mutant, which lacks all CWAs on the cell surface, had the lowest expression of cytokines. It is important to note that the sortase A mutant still makes all the CWAs, but they are secreted into the environment instead of covalently attached to the cell wall. However, it does suggest that the influence on immune signaling is greatest when the CWAs are still attached to the bacteria. However, more testing would be needed in defining the portions of each CWA responsible for altering immune signaling.

During our screen we found that the *sasD*::*Tn* mutant had decreased levels of myeloid cytokines and increased gene expression of lung homeostatic markers in bacterial pneumonia. We found similar findings with a transduced mutant. Most striking was the delay in mortality in mice infected with sasD A50.1, which may be explained by the decrease in bacterial survival seen with sasD A50.1 infection compared to the WT strain. What could be causing this decrease in bacterial survival is unknown, as this protein is uncharacterized. Complementation with SasD increased bacterial burden and BAL cells to WT levels, suggesting that this protein is causing the observed phenotype. While it is known that SasD has a punctate surface expression versus a ring-like distribution of most CWAs (50, 51), it is unclear if this is contributing to the differences seen in our model. This decrease in bacterial survival of sasD A50.1 could explain the inflammatory differences seen early and late during bacterial pneumonia.

At 6 h postinfection, there is a significant reduction in the macrophages in the lung, as well as IL-1β. It is known that macrophage-derived IL-1β can induce excessive inflammation and pathology in the lung (52, 53). The reduction in IL-1β could be explained by the decrease in macrophages early during infection. This decrease in inflammation continued at 24 h postinfection, where there was a reduction in the levels of neutrophils, which can cause excessive damage themselves (54). While we did not see functional changes in neutrohphils via qPCR, we did see a decrease in the neutrophil:eosinophil ratio within the lung at 24 h postinfection with sasD A50.1. Eosinophils have been implicated in antibacterial immunity (55), and the increased ratio of eosinophils could help control the bacterial burden in the lung. It has been shown that IL-33 induction of type 2 responses is protective in lethal models of S. aureus sepsis and pneumonia by counterbalancing proinflammatory responses (56, 57). While we did not see any differences in IL-33 (data not shown) or gross pathology at 24 h postinfection, we did see a reduction in type 17 cytokines and neutrophils, which has been shown to be protective in patients with S. aureus infection (57, 58). Thus, the reduction in inflammation or alteration of inflammatory cell ratios could help explain the delayed mortality seen in mice.

Since we saw a change in IL-1β production both early and late during infection, we decided to examine the inflammasome. S. aureus is known to prime and activate the NLRP3 inflammasome via pore-forming toxins, such as the alpha-toxin (26). The NLRP3 inflammasome activates caspase 1, which cleaves pro-IL-1β (30). However, we did not see any difference in alpha-toxin activity via blood hemolysis between strains (data not shown). We did see a significant downregulation of *il-1β* and *nlrp3* transcripts but not the more common ASC (*pycard*) component, suggesting that, potentially, the priming step of the NLRP3 inflammasome expression may be reduced. Priming of the NLRP3 inflammasome is thought to be due to sensing of S. aureus lipoproteins and Toll-like receptor (TLR)-2 and -4 signaling (26, 59). While we did not see changes in expression in TLR-2 or -4 in macrophages (data not shown), we cannot rule out the possibility that SasD may be involved in the sensing of S. aureus. When infected with sasD A50.1, RAW264.7 cells had a reduction in *il-1β* and *tnfα* without a significant change in bacterial burden or bacterial phagocytosis. In BMDMs, we saw a similar trend in gene expression and also saw increased viability when they were infected with sasD A50.1 compared to WT S. aureus. While we did not see any differences in pro-IL-1β or caspase 1 activity at 3 h postinfection, there may be other cell death pathways involved, such as necroptosis. Blocking necroptosis has been shown to reduce bacterial burden and damage during S. aureus pneumonia (29), similar to the sasD A50.1 *in vivo* phenotype, and can feed into NLRP3 inflammasome and pyroptosis induction (60). Thus, the decrease in IL-1β could be due to changes in cell death pathways that funnel into the NLRP3 inflammasome in macrophages.

In conclusion, we identified a critical role for SasD in bacterial pneumonia associated with increased bacterial burden, inflammation, and mortality. SasD may contribute to survival of S. aureus in the lung, as there is decreased bacterial survival in the mutant at both 6 and 24 h postinfection. SasD promotes induction of early IL-1β production in macrophages, which consequentially recruit neutrophils into the lung at later time points, leading to increased inflammation. These data suggest that early targeting of SasD in the lung may reduce future inflammation signaling during staphylococcal pneumonia.

## MATERIALS AND METHODS

### Mice.

Six- to eight-week-old male and female WT C57BL/6Ntac mice were purchased from Taconic Farms. Mice were maintained under pathogen-free conditions within the animal facilities at the UPMC Children’s Hospital of Pittsburgh. All studies were performed on sex- and age-matched mice. Animal studies were conducted with approval from the University of Pittsburgh Institutional Animal Care and Use Committee.

### S. aureus strains.

USA300 multidrug-resistance S. aureus (MRSA) strain JE2 (61) was the WT strain for all studies. All strains used in study are listed in Table S1 and are derived from the Nebraska Transposon Mutant Library (61) (BEI Resources), with strains *srtA*::*Tn*, *sdrE*::*Tn*, *sdrC*::*Tn*, *fnbB*::*Tn*, *isdB*::*Tn*, *sasG*::*Tn*, and *sasD*::*Tn* given by Ken Urish, University of Pittsburgh. All mutants were confirmed by PCR using the gene- and transposon-specific primers listed in Table S2. Strain sasD A50.1 was generated via phage 11 transduction of *sasD*::*Tn* lysate into the wild-type JE2 strain, selected with 5 μg/mL erythromycin and confirmed by PCR (Table S2). The sasD complemented strain (sasD A50.1 + pSasD) was created by cloning the *sasD* locus (Table S2) into the shuttle vector pOS1-P_lgt_ (62) with a constitutive promoter using FastDigest BamHI and XhoI restriction enzymes (Thermo Scientific). The resulting plasmid was cloned into One Shot TOP10 Escherichia coli (Invitrogen), selected with 100 μg/mL ampicillin, and harvested using the GeneJET plasmid miniprep kit (Invitrogen). The plasmid was electroporated into S. aureus restriction-deficient strain RN4220 (63), selected with 20 μg/mL chloramphenicol, and the resulting lysate was transduced via phage 11 into strain sasDA50.1. S. aureus strains were grown in tryptic soy broth (BD Bacto) overnight at 37°C at 250 rpm. Overnight cultures were diluted 1:100 and grown to an optical density at 660 nm (OD_660_) of ~1, approximating the logarithmic growth phase. The MRSA dose was calculated using the OD_660_ measurement of the culture and application of a calculated extinction coefficient. For growth curves, overnight cultures were diluted 1:200 in a 96-well plate in sexaplicate. Plates were grown at 37°C at 282 rpm in a Synergy H1 hybrid multimode reader (BioTek). Optical density measurements at 660 nm were taken every 30 min. Growth rate (μ) was calculated from at least two independent experiments using the equation A_t_ = A_t–1_ · e^μt^. The μ_max_ was calculated as the average of the three highest μ rates.

### Murine models.

Influenza A/PR/8/34 (H1N1) was grown in chicken eggs as previously described (64). Mice were inoculated with phosphate-buffered saline (PBS) vehicle or 100 PFU of influenza in 50 μL of sterile PBS. Six days later, mice were infected with 1 × 10^8^ CFU of MRSA in 50 μL of sterile PBS. All infections were performed via oropharyngeal aspiration. Mice were harvested 6 or 24 h after MRSA challenge using pentobarbital injection (300 mg/kg of body weight) and cervical dislocation. In mortality studies, a dose of 2 × 10^8^ CFU was used. During harvest, the lung was lavaged with 1 mL sterile PBS. BAL cells were pelleted, and red blood cells were lysed (ACK lysis buffer, Gibco). Cells were resuspended, placed on slides via cytospin, stained with Hema 3 (Thermo Fisher), and quantified. The right upper lung lobe was homogenized in 1 mL PBS and plated on tryptic soy agar for determination of bacterial burden. The remaining right lung was frozen in liquid nitrogen and stored at −80°C for gene expression analysis. The left lobe was perfused with 10% formalin and embedded in paraffin. Lung sections were stained with hematoxylin and eosin, and inflammatory features were evaluated via microscopy after sample blinding. For competitive index studies, mice were inoculated with a 1:1 ratio of JE2 and sasD A50.1 strains at a total of 1 × 10^8^ CFU. Whole lungs were homogenized in 2 mL of sterile PBS and plated on tryptic soy agar with and without erythromycin (5 μg/mL). The competitive index was calculated as the ratio of sasD A50.1:JE2 CFU at sacrifice divided by the ratio at the time of inoculation.

### Macrophage experiments.

RAW264.7 cells were used, and BMDMs were isolated as previously described (65). For experiments, 7 × 10^5^ cells were plated in 6-well plates, infected at a multiplicity of infection (MOI) of 10 (RAW264.7) or 50 (BMDMs) and spun at 250 × *g* for 5 min at 4°C to synchronize infection. For RAW264.7 experiments, cells were infected for 1 h in the absence of antibiotics, and media\um was replaced with antibiotic- and serum-free medium with and without gentamicin (100 μg/mL) for 1 h, and then replaced with antibiotic-free medium for an additional hour. At collection, cells were lysed with 1% Triton X-100 at room temperature for 10 min, and 50 μL was collected for CFU determination. Phagocytosis was calculated by the equation ([CFU + gentamicin]/[CFU – gentamicin]) · 100. RLT (Qiagen) was added to the wells and collected and run through a Qiashredder and frozen at −80°C until RNA extraction. For BMDM experiments, cells were left to rest overnight and treated with 10 ng/mL IFN-γ (R&D Systems) for 24 h. BMDMs were infected for 3 h, washed, and resuspended in antibiotic-free RPMI medium. BMDM viability was determined using trypan blue (Gibco) staining and the Countess 3 automatic cell counter (Invitrogen). BMDMs wells were combined and incubated in RIPA buffer (25 mM Tris, 150 mM NaCl, 1% NP-40, 0.1% SDS, 5 mM EDTA, 0.5% sodium deoxycholate) for 30 min at 4°C with agitation, centrifuged at 10,000 rpm for 10 min at 4°C, and frozen at −80°C until Western blotting was done. Primary antibodies were rabbit anti-IL-1β (Abcam 254360), rabbit anti-caspase 1 (Abcam 138483), rabbit anti-caspase p20 (Invitrogen PA5-99390), and mouse anti-β-actin (Cell Signaling 8H10D10). Samples were thawed, proteins were quantified using bicinchoninic acid (BCA) protein assay (Pierce), boiled in Laemmli buffer (Bio-Rad), and loaded on a 4 to 20% gel (Bio-Rad). Proteins were transferred to a polyvinylidene difluoride (PVDF) membrane using the Trans-Blot Turbo transfer system (Bio-Rad). Blots were probed with primary antibodies and donkey anti-mouse or goat anti-rabbit secondary antibodies conjugated to IRDye 800CW or 680RD fluorophores (LI-COR). Blots were imaged using the Odyssey CLx and analyzed using Image Studio (LI-COR). Relative protein expression is normalized to beta-actin levels in each sample.

### RNA extraction and qPCR.

RNA was extracted from mouse lungs using the Absolutely Total RNA purification kit (Agilent). RNA extraction from cell culture experiments was performed using the Qiagen RNeasy kit (Qiagen). RNA was quantified and converted to cDNA using the iScript cDNA synthesis kit (Bio-Rad). Quantitative PCR was performed using SsoAdvanced universal probes supermix (Bio-Rad) and TaqMan primer-probe sets (Thermo Fisher Scientific) listed in Table S3 on the CFX96 Touch real-time PCR detection system (Bio-Rad). Gene expression was calculated using the ΔΔ*CT* method using *hprt* as a housekeeping gene and normalized to the average WT S. aureus values unless otherwise stated.

### Multiplex and heatmap analysis.

Lung homogenate cytokines were assessed using the Bio-Plex Pro mouse cytokine 23-plex assay (Bio-Rad). For clustering analysis, all data were combined and samples with missing data and macrophage inflammatory protein-1α (MIP-1α), due to poor detection, were excluded. The average for each cytokine per mutant was used. Using R (version 4.1.0) in RStudio (version 1.4.1717), data were log-transformed and scaled to the Z score and clustered by cytokine using the hclust function and Pearson correlation. The resulting heatmap was visualized using the Heatmap.2 function in the gplots package.

### Statistical analysis.

Data were analyzed using Prism 8 (GraphPad). Analyses comparing two groups were performed using the Mann-Whitney test or an unpaired *t* test. For analyses assessing more than two groups, the Kruskal-Wallis test with Dunn’s multiple-comparison correction was used. Analyses comparing two variables were tested via two-way analysis of variance (ANOVA) with Sidak’s multiple-comparison correction. Mortality data were analyzed by a log rank (Mantel-Cox) test. All figures show combined data from multiple replicate studies and are graphed as the mean ± standard error of the mean (SEM). *N* values are numbers of animals per independent experiment. Statistical significance (*P* ≤ 0.05) is indicated in the figure legends, with *P* values between 0.05 and 0.1 displayed numerically.
