# Supplementary material for: Novel Requirement for Staphylococcal Cell Wall-Anchored Protein SasD in Pulmonary Infection
Source: Microbiol Spectr. 2022 Aug 30;10(5):e01645-22. doi: 10.1128/spectrum.01645-22 (PMC9603976; doi:10.1128/spectrum.01645-22)
Supplement: Supplemental file 1 — Download spectrum.01645-22-s0001.pdf, PDF file, 0.6 MB [file spectrum.01645-22-s0001.pdf]

Supplemental Table 1: *S. aureus* strains used in this study

Strain	Description	NTML #	Reference
JE2	NTML Wildtype strain		(61)
JE2 *srtA::Tn*	NTML mutant	NE1787	(61)
JE2 *clfA::Tn*	NTML mutant	NE543	(61)
JE2 *clfB::Tn*	NTML mutant	NE391	(61)
JE2 *fnbB::Tn*	NTML mutant	NE728	(61)
JE2 *sdrC::Tn*	NTML mutant	NE432	(61)
JE2 *sdrD::Tn*	NTML mutant	NE1289	(61)
JE2 *sdrE::Tn*	NTML mutant	NE98	(61)
JE2 *isdB::Tn*	NTML mutant	NE1102	(61)
JE2 *sasG::Tn*	NTML mutant	NE825	(61)
JE2 *sasD::Tn*	NTML mutant	NE1032	(61)
sasD A50.1	Transduced strain from JE2 *sasD::Tn*		This study
RN4220	Methicillin Sensitive Restriction Deficient *S. aureus*		(63)
sasD A50.1 + pSasD	Complemented sasD A50.1 strain		This study

Supplemental Table 2: *S. aureus* primers used

Strain	Primer (5’-3’)	Source	Transposon Primer Used
JE2 *srtA::Tn*	CCAAACGCCTGTCTTTTCAT	This study	Buster
JE2 *clfA::Tn*	AAACACGCAATTCGGAAAAA	This study	Buster
JE2 *clfB::Tn*	TTCGCACTGTTTGTGTTTGC	This study	Upstream
JE2 *fnbB::Tn*	CTCCCGCCTTAATTCCTTCT	This study	Upstream
JE2 *sdrC::Tn*	CAATATTTCCGTCCAGGATCA	This study	Buster
JE2 *sdrD::Tn*	CAAAAAGGTAGATGCCAAAACTG	This study	Upstream
JE2 *sdrE::Tn*	CCATCAGGAGAGGTCATTGC	This study	Buster
JE2 *isdB::Tn*	CAAACCAACACCATCACCTG	This study	Buster
JE2 *sasG::Tn*	TGGAAAGTTTCATGGGCAAC	This study	Buster
JE2 *sasD::Tn*	CATGCCGACACAACTTCAAT	This study	Upstream
sasD A50.1	CATGCCGACACAACTTCAAT	This study	Upstream
sasD A50.1 + pSasD	SasD For:AGCACGCTCGAGATGAAAAAATTAGCAACAGTA	This study	
	SasD Rev: TCGACTGGATCCTTATTTATTTGTTGAACGTCG	This study	

Supplemental Table 3: TaqMan Primer-Probes used in study

Gene	Catalog Number
IL-17a	Mm00439618_m1
IL-23a	Mm00518984_m1
Cxcl1	Mm04207460_m1
IL-1β	Mm00434228_m1
NLRP3	Mm00840904_m1
Pycard	Mm00445747_g1
Foxj1	Mm01267279_m1
TJP1	Mm00493699_m1
Cav1	Mm00483057_m1
Sftpc	Mm00488144_m1
Scgb1a1	Mm00442046_m1
Muc5b	Mm00466376_m1
MPO	Mm01298424_m1
ELANE	Mm00469310_m1
CTSG	Mm00456011_m1

Supplemental Figure Legends

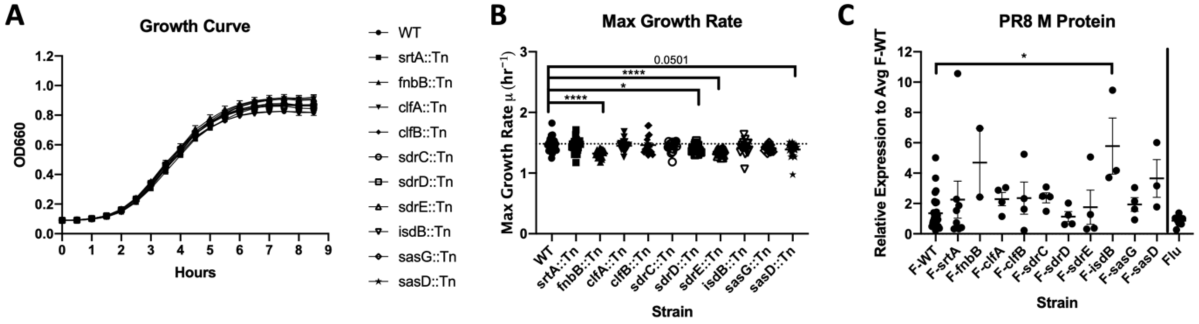

Supplemental Figure 1: Bacterial Growth and Viral Burden. A. WT or mutant MRSA (see graphs) were grown overnight in tryptic soy broth and diluted 1:200 in a 96-well microtiter plate in sexaplicate. Plates were grown at 37°C at 282 rpm continuously. Measurements at 660 nm were taken every 30 minutes. Combination of at least 2 experiments per strain. **B.** Max growth rate of WT or mutant MRSA strains (see graphs). Growth rate (μ) was calculated off at least two independent experiments using the equation $A_t=A_{t-1}*e^{\mu t}$ (see methods). The μ_{max} was calculated as the average of the three highest μ rates. **C.** Relative expression of influenza PR8 M protein via qPCR normalized to the average F-WT values. Statistics tested by Kruskal-Wallis with Dunn's multiple comparisons correction (B-C). * p<0.05, ****p<0.0001. N=2-6, combination of several experiments, data graphed as mean ± SEM. srtA: Sortase A, fnbB: fibronectin binding protein B, clfA/B: clumping factor A/B, sdrC/D/E: serine-aspartate repeat containing protein C/D/E, isdB: iron-regulated surface determinant B, sasD/G: *S. aureus* surface protein D/G.

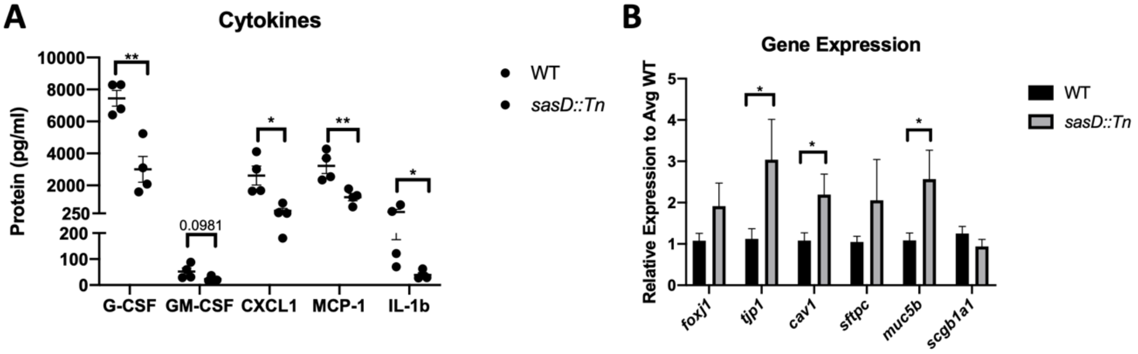

Supplemental Figure 2: SasD Increases Inflammatory Cytokine and Decreases Lung Homeostatic Gene Expression. A. Lung homogenate protein levels of cytokines in mice infected with WT or *sasD::Tn* MRSA during bacterial pneumonia. **B.** Gene expression of lung epithelial markers in mice infected with WT or *sasD::Tn* MRSA during bacterial pneumonia. Statistics tested by unpaired t test. * $p<0.05$, ** $p<0.01$. N=2, combination of several experiments, data graphed as mean ± SEM.

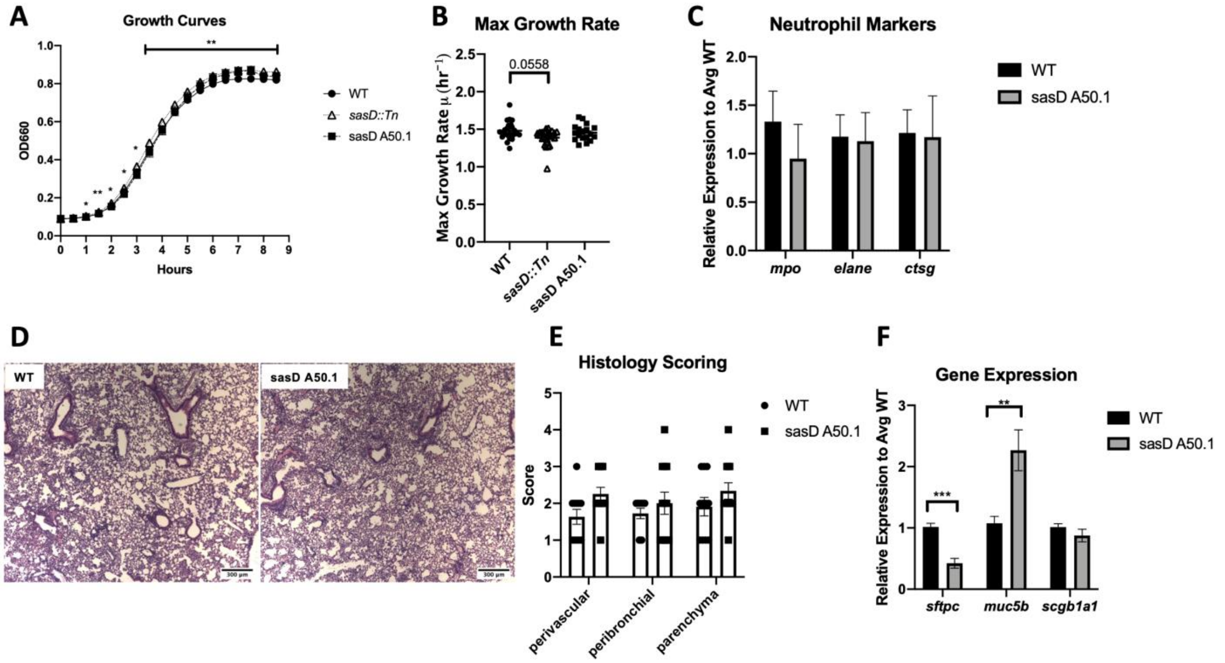

Supplemental Figure 3: Characterization of SasD during Pneumonia. A. WT or mutant MRSA (see graphs) were grown overnight in tryptic soy broth and diluted 1:200 in a 96-well microtiter plate in sexaplicate. Plates were grown at 37°C at 282 rpm continuously. Measurements at 660 nm were taken every 30 minutes. Combination of at least 2 experiments per strain. Statistical significance is between WT and *sasD::Tn* MRSA strains. **B.** Max growth rate of WT or mutant MRSA strains (see graphs). Growth rate (μ) was calculated off at least two independent experiments using the equation $A_t=A_{t-1}*e^{\mu t}$ (see methods). The μ_{max} was calculated as the average of the three highest μ rates. **C.** Gene expression of neutrophil markers relative to the average WT values. **D.** H&E-stained lung sections infected with WT or sasD A50.1 for 24 hours **E.** Histology scoring of lung sections. **F.** Gene expression of lung epithelial markers relative to the average WT values. Statistics done by Mixed-effects model with Dunnett's multiple comparisons correction (A), Kruskal-Wallis Test with Dunn's multiple comparisons correction (B), unpaired T test (C, E-F). * $p<0.05$, ** $p<0.01$, *** $p<0.001$, N=2-6, combination of multiple experiments, data graphed as mean ± SEM.

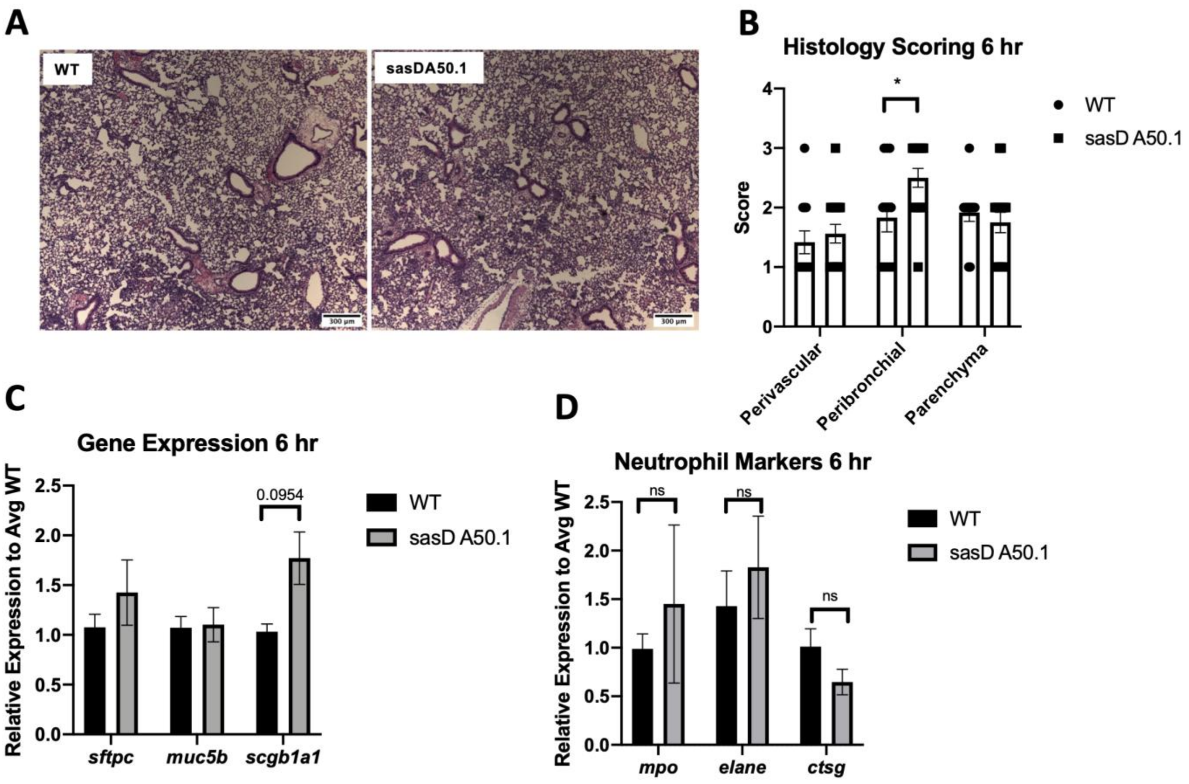

Supplemental Figure 4: Characterization of early SasD infection. A. H&E-stained lung sections infected with WT or sasD A50.1 for 6 hours **B.** Histology scoring of H&E-stained lung sections. **C-D.** Gene expression of lung epithelial (**C**) and neutrophil (**D**) markers relative to the average WT values. Statistics done by Two-way ANOVA with Sidak's multiple comparisons correction (B), unpaired T test (C-D). * $p<0.05$, n=4-6, combination of multiple experiments, data graphed as mean ± SEM.
